# Knowledge, attitude, and practice of preconception care and associated factors among obstetric care providers working in public health facilities of West Shoa Zone, Ethiopia: A cross-sectional study

**DOI:** 10.1371/journal.pone.0272316

**Published:** 2022-08-01

**Authors:** Hawi Abayneh, Negash Wakgari, Gemechu Ganfure, Gizachew Abdissa Bulto

**Affiliations:** 1 Departmentof Midwifery, College of Medicine and Health Sciences, Salale University, Fitche, Ethiopia; 2 Department of Midwifery, College of Medicine and Health Sciences, Ambo University, Ambo, Ethiopia; 3 Department of Pediatrics and Child Health, College of Medicine and Health Sciences, Ambo University, Ambo, Ethiopia; VART Consulting PVT LTD, INDIA

## Abstract

Preconception care is biomedical, social, and behavioural care provided for a woman or couple before conception occurs or throughout their reproductive year. In Ethiopia, it’s reported that the majority of health care providers had poor knowledge and practice of preconception care. The institution-based cross-sectional study was conducted among 359 obstetric care providers to assess knowledge, attitude, and practice of preconception care in West Shoa Zone, Ethiopia. A stratified, simple random sampling technique selected five hospitals, 46 health centers, and study participants. Pretested and structured questionnaires were used to collect data. Data were entered into Epidata and exported to SPSS for analysis. Bivariate and multivariate logistic regressions were employed to identify an association between the independent predictors and the outcome variables. In this study, 173(48.2%) and 124(34.5%) of the obstetric care providers had good knowledge and practice of preconception care, respectively. Two-thirds 255(71%) of providers had a favorable attitude toward preconception care. The odds of having good knowledge were higher among Midwives’ providers [AOR: 2.03, 95%CI: 1.09–3.77] and had training on HIV testing [AOR: 3.5, 95%CI: 1.9–6.4]. The presence of a library [AOR: 1.7, 95%CI: 1.04–2.85] and internet access [AOR: 3.4, 95%CI: 2.0–5.8] in working health facility had a higher odds of good knowledge about preconception. Degree and above holders [AOR: 3.1, 95%CI: 1.5–6.1] also had higher odds of good preconception knowledge than diploma holders. Similarly, the odds of having good practice of preconception care were higher among health care providers: who did screening for reproductive life plans [AOR: 3.7, 95%CI:1.8–7.4], worked in maternity and child health unit [AOR:4.2,95%CI:2.0–8.6], perceive all health facilities should give preconception care services [AOR:2.3,95%CI:1.2–4.3], and perceive all health care providers should provide preconception services [AOR:3.0, 95%CI: 1.7–5.5]. This study found that more than half of obstetric care providers’ had poor knowledge, favorable attitude, and poor practice of preconception care. Provision of training, carrier development, and installation of internet and library services should be enhanced.

## Introduction

World Health Organization (WHO) defines Preconception care as “biomedical, social, and behavioural care provided for a woman or couples before conception occurs or throughout their reproductive year.” To bring a successful preconception care initiative, in 2013, the world health organization (WHO) developed preconception care packages that encompass 13 areas to be addressed by the health care providers [1]. Obstetricians and other obstetric care providers should engage each patient in supportive, respectful conversation about the women’s pregnancy intentions and provide pre-pregnancy or contraceptive counseling based on the women’s desires and preferences [2].

Study shows that in 2017 global maternal mortality ratio was 211 per 100,000 live births [3], and other evidence indicated that in 2019 the neonatal mortality rate was 17 per 1000 live births [4]. In Ethiopia, the maternal mortality ratio was 412 maternal deaths per 100,000 live births, while under-five children’s, infants, and neonatal mortality rates were 67, 45 and 29 per 1000 live births, respectively [5]. Reducing maternal mortality depends on ensuring that ladies have access to quality care before, during, and after childbirth [6]. However, in low and middle-income countries, many women do not have adequate access to the prenatal care they need [7].

The likelihood of using preconception care (PCC) services is dependent on health care workers’ knowledge, attitudes, and providing information on preconception care (PCC) to their patients [8]. However, a study done in West Shoa Zone, Oromia, showed that 8.1% of women got information and counseling from the health care providers (HCPs) and 14.5% of them utilize the service [9]. This finding suggests that healthcare providers (HCPs) may have a crucial influence on couples’ use of PCC. HCPs have an obligation and take the top place to update evidence-based clinical practices associated with preconception care and have updated knowledge to give PCC [10].

In Ethiopia, some studies show that more than half of healthcare providers have poor knowledge [11–13], and more than 85% of healthcare providers are practicing poorly [14]. Moreover, previous studies have identified different factors for health care providers’ knowledge and practice on preconception care, including socio-demographic characteristics, training, attitude towards PCC, availability of policy or protocol on PCC, availability of internet access and library in the working facility [11–14]. Even though some studies were conducted in some parts of Ethiopia on the HCPs’ knowledge and practice, the studies were conducted on total health care providers rather than obstetric care providers (OBCPs) [11–15]. There is a lack of information on knowledge, attitude, and practice of preconception care among obstetric care providers in West Shoa Zone, Oromia. As a result, this study intended to assess the Knowledge, attitude, and practice of preconception care and associated factors among obstetric care providers in public health facilities of West Shoa Zone, Oromia, Ethiopia.

## Methods and materials

### Study design, setting and population

An institution-based cross-sectional study was conducted in West Shoa Zone, Oromia regional state, from August 1 to September 8, 2021. West Shoa Zone is one of the zones in the Oromia Region of Ethiopia. Its administrative city is Ambo town, located 114 kilometers away from the capital city of Ethiopia, Addis Ababa. The West Shoa Zone has 9 Public Hospitals, 92 health centers, and 529 health posts. About 1,085 obstetric care providers were working in maternity and reproductive care units of the Zone. Among those 11 were Obstetricians and Gynecologists, 21 Integrated Emergency Surgical Officers (IESO), 38 General Practitioners, 426 Midwives, 467 nurses, and 222 Health Officers. About 268 OBCPs worked in hospitals during the data collection period, while 817 worked in Health Centers.

All obstetric care providers working in the public health facilities of West Shoa Zone during the study period were considered the source population. In contrast, all obstetric care providers working in West Shoa Zone’s selected public health facilities were considered the study population. In addition to this, all obstetric care providers working in public health facilities of West Shoa Zone during the data collection period were included in the study, and those OBCPs who served for less than six months were excluded from the study.

### Sample size determination and sampling procedure

The sample size for the first two specific objectives: to determine knowledge and preconception care practice among obstetrics care providers, was calculated by a single population proportion formula n = (Z α/2)2 P (1-P)/d2 based on the following assumptions: the proportion (P) of knowledge and practice were 31% [11] and 19.2% [15] respectively which was taken from the previous studies, 95% confidence level of Z α/2 = 1.96, 5% of absolute precision. Thus, a 10% non-response rate gave 362 and 262, respectively, and the final sample size became 362.

The facilities were stratified into hospitals and health centers. With a simple random sampling technique, 5(N = 157) hospitals and 46(N = 401) health centers were selected. The sample was allocated to each stratum proportionally based on the number of health care providers working at the selected hospitals and health centers, 157/558*362 = 102 OBCPs from hospitals and 401/558*362 = 260 OBCPs from health centers. Then, a simple random sampling technique was used to select study participants.

### Data collection tools and procedures

The data were collected using structured self-administered questionnaires. The questionnaire was adapted from a previous study conducted in Ethiopia [11] and had five sections (socio-demographic information, knowledge, attitude and practice, and associated factors of preconception care questions). The knowledge section had 15 knowledge-related questions. A 1 and 0 were given for correct and incorrect answers, respectively. Then, HCPs who scored ≥ 50th percentile were considered good knowledge of PCC.

The practice section had 34 questions with three PCC practice components measuring the frequency of practice in the last six months of the period. Each question have an option response of never = 0, rarely = 1, sometimes = 2, often = 3, and always = 4. This gives a minimum score of 34*0 = 0 to the maximum score of 34*4 = 136. In this study, those OBCPs who scored ≥ 50% were considered good PCC practice; otherwise poor.

Similarly, the OBCPs’ attitudes were assessed by their level of agreement on nine questions using the Likert scale. Those OBCPs who scored 60% and above the possible maximum score were considered OBCPs with a favorable attitude towards PCC; otherwise, they were considered OBCPs with an unfavorable attitude toward PCC. The questionnaires were disseminated to the OBCPs and facilitated by six trained graduated unemployed nurses and supervised by two senior BSc midwives.

### Data quality assurance

To assure quality of the study, data collectors and supervisors were given two days of training about the study materials and data collection procedures. Before actual data collection, the study tool was pre-tested among 18 OBCPs working at Tulu Bolo hospital in South West Shoa Zone, Ethiopia. The tool’s reliability was checked for its internal consistency with a Cronbach’s α test, 0.802 and 0.97 for knowledge and practice, respectively. Moreover, the completeness and consistency of the collected data were reviewed and checked by supervisors and investigators.

#### Operational definition

*Obstetric care providers*. Certified obstetricians and gynecologists, general practitioners, integrated emergency surgical officers, nurses, midwives, and public health officers working in maternal and reproductive health care units [16].

*Knowledge of PCC*. Respondents who scored less than the 50th percentile of the knowledge-related items were categorized as HCPs with `poor PCC knowledge.’ In contrast, HCPs who scored ≥ 50th percentile were considered good knowledge of PCC [12].

*Attitude towards PCC*. Attitude was measured using nine questions with possible five-point Likert scale responses. Those OBCPs who scored 60% and above the possible maximum score [5*9 = 45] were considered OBCPs with a favorable attitude towards PCC; otherwise, they were considered OBCPs with unfavorable attitudes towards PCC.

*OBCP’s PCC practice*. OBCPs who scored < 50% of PCC practice items were classified as practitioners demonstrating poor PCC practice. Those OBCPs who scored ≥ 50% were considered good PCC practice [14].

### Data analysis procedures

The collected data were cleaned, coded, and entered into the Epidata version 3.1 and exported to SPSS version 20. Coding was reversed in negative statements. The frequency, proportion, mean, and standard deviation were performed for dependent and independent variables.

Binary logistic regression was done to identify candidate variables for multiple logistic regressions. Then those candidate variables were analyzed with multivariate logistic regression, and those variables at a p-value of < 0.05 were considered to have a statistically significant association with the outcome variables. Odds ratios with 95%CI were used to test the strength of association. Multicollinearity assumption was checked by the variance inflation factor (VIF) of < 1.2, which indicated a less likely correlation between independent variables. The models’ goodness of fit was tested using the Hosmer-Lemeshow test, and it was a good fit.

### Ethics statement

The ethical approval was obtained from Ambo University ethical review board. Written Informed consent was obtained from each participant. The participants were assured that participation in the study was voluntary and that they could withdraw during the study. The collected raw data was kept confidential in a secure place, and the names of the participants were not written in the study record. Participants’ rights to anonymity and confidentiality were fully protected. All of the information given by participants was recorded in a manner that did not link the respondents with the data.

## Results

### Socio-demographic characteristics of the respondents

Out of the total samples, 359 obstetric care providers participated with a 99% response rate. More than half, 183(51%) of the participants were male. Of all participants, 145(40.4%) werefound between 26–30 years old. More than half, 207(57.7%) of respondents had more than five years of service experience, and 285(79.4%) had degreesand above (Table 1).

**Table 1:** Obstetric care providers’ socio-demographic characteristics in West Shoa Zone, Oromia, Ethiopia, 2021(n = 359).

Variables		Frequency	Percentage
Gender	Male Female	183 176	51.0 49.0
Age group in years	20–25 26–30 31–35 ≥36	17 145 97 100	4.7 40.4 27.0 27.9
Marital status	Single Married Divorced Widowed Living together	119 188 30 8 14	33.1 52.4 8.4 2.2 3.9
Profession	Midwives Nurses General practitioners Public health officers Others*	168 88 27 66 10	46.8 24.5 7.5 18.4 2.8
Working institution	Hospital Health center	102 257	28.4 71.6
Working experience inyears	≤ 5	152	42.3
	>5	207	57.7
Educational level	Diploma Degree and above	74 285	20.6 79.4
Working unit	MCH Gynecologic OPD Gynecologic ward Labor and deliveryward	108 67 51 133	30.1 18.7 14.2 37.0

### Obstetric care provider’s knowledge about preconception care

Out of the total participants, 173(48.2%) [95%CI: 43.2–53.5] of OBCPs had a good knowledge of preconception care. The knowledge score range from 1to 12. The majority, 233(64.9%), knew that the eligible clients for PCC include all adolescents and reproductive age individuals (Table 2).

**Table 2:** Obstetric care providers’ knowledge about preconception care in West Shoa Zone, Oromia, Ethiopia, 2021(n = 359).

Variables	Response category	Frequency	Percentage
The eligible clients for PCC include all adolescents and reproductive age individuals	Yes	233	64.9
	No	122	34.0
	Do not know	4	1.1
To be effective, PCC should start four weeks before conception	Yes	190	52.9
	No	162	45.1
	Do not know	7	1.9
Periodontal disease is a risk factor for adverse pregnancy outcomes (APO)	Yes	57	15.9
	No	129	35.9
	Do not know	173	48.2
Planning pregnancy with a BMI of ≤ 18.4 increases the risk of developing APO	Yes	286	79.7
	No	43	12.0
	Don’t know	30	8.4
All women of reproductive age should take 400 mcg of folic acid daily	Yes	255	71.0
	No	83	23.1
	Do not know	21	5.8
Women need to start taking folic acid 3months before pregnancy	Yes	77	21.4
	No	211	58.8
	Do not know	71	19.8
The recommended routine preconception laboratory tests include Hct, HIV,HBV, and RPR or VDRL tests	Yes	79	22.0
	No	208	57.9
	Do not know	72	20.1
Preconception genetic counseling and screening include recommending carrier screening tests for the client with sickle cell hemoglobinopathies	Yes	93	25.9
	No	234	65.2
	Do not know	32	8.9
Isotretinoin, Valproic acid, and Warfarin are medications that pose teratogenic effects requiring preconception modification	Yes	120	33.4
	No	150	41.8
	Do not know	89	24.8
Early identification and treatment of diseases like depression, seizure disorder, and phenylketonuria during the preconception period reduce the occurrence of APO	Yes No	117	32.6
		179	49.9
	Do not know	63	17.5
The recommended test that guarantees good preconception blood sugar control for a woman with pregestational diabetes is the random blood sugar test	Yes	121	33.7
	No	217	60.4
	Do not know	21	5.8
Recommending regular exercise is an important PCC counseling point. Thus, women planning pregnancy should aim for 30 minutes of moderate exercise 5 days a week	Yes	110	30.6
	No	183	51.0
	Do not know	66	18.4
Women planning pregnancy should be advised to delay pregnancy until reducing drug, alcohol, and tobacco use	Yes	218	60.7
	No	129	35.9
	Do not know	12	3.3
A clinician attending to clients with previous caesarian section should advise the client to delay the next pregnancy for at least 18 months before the next conception	Yes	285	79.4
	No	66	18.4
	Do not know	8	2.2
Infertility screening and management is not the concern of PCC	Yes	243	67.7
	No	67	18.7
	Do not know	49	13.6

### Attitude toward preconception care

Two-thirds, 255(71%) of OBCPS had a favorable attitude towards PCC. Out of the total 45 scores, respondents scored a minimum of 12 and a maximum of 41, with a mean score of 28.1 with SD ± 4.96. More than half, 216(60.2%) of the respondents strongly disagreed that providing PCC is not within the scope of my professional responsibility and accountability. About one-third, 127(35.5%) of respondents strongly agreed with the idea that omission of preconception care leads to irreversible damage to the fetus (Table 3).

**Table 3:** Obstetric care providers’ attitude towards preconception care in West Shoa Zone, Oromia, Ethiopia, 2021(n = 359).

Variables	Response category	Frequency	Percentage
Omission of preconception care leads to an irreversibledamage to the fetus	Strongly disagree	125	34.8
	Disagree	36	10
	Undecided	26	7.2
	Agree	45	12.5
	Strongly agree	127	35.5
PCC provides the most incredible opportunity to optimize couples’ health particularly women’s health before conception	Strongly disagree	120	33.4
	Disagree	32	8.9
	Undecided	21	5.8
	Agree	45	12.5
	Strongly agree	141	39.3
Providing PCC services to developing countries like Ethiopia is a luxury service	Strongly disagree	90	25.1
	Disagree	139	38.7
	Undecided	64	17.8
	Agree	42	11.7
	Strongly agree	24	6.7
In developing countries like Ethiopia, the focus of PCC should not be directed to healthy people but to people with infectious diseases like HIV and HBV	Strongly disagree	201	56
	Disagree	62	17.3
	Undecided	34	9.5
	Agree	42	11.7
	Strongly agree	20	5.6
Providing PCC is not within the scope of my professional responsibility and accountability	Strongly disagree	216	60.2
	Disagree	69	19.2
	Undecided	33	9.2
	Agree	31	8.6
	Strongly agree	10	2.8
Due to the presence of other competing demands, providing PCC is not the priority intervention I should provide	Strongly disagree	203	56.5
	Disagree	72	20.1
	Undecided	39	10.9
	Agree	32	8.9
	Strongly agree	13	3.6
Preconception care should be given for all healthy and sick individuals including those presented with a critical and emergency condition	Strongly disagree	224	62.4
	Disagree	75	20.9
	Undecided	31	8.6
	Agree	24	6.7
	Strongly agree	5	1.4
All healthcare providers can easily integrate the elements of PCC in their daily practice to all eligible individuals whom they are caring	Strongly disagree	144	40.1
	Disagree	70	19.5
	Undecided	60	16.7
	Agree	24	6.7
	Strongly agree	61	17
Preconception health is part of the reproductive andthe human rights issue to which the health professional is responsible either for omission or commission of PCC	Strongly disagree	186	51.8
	Disagree	108	30.1
	Undecided	23	6.4
	Agree	27	7.5
	Strongly agree	15	4.2

### Preconception care practice among health care providers

Out of the total participants, 124(34.5%) of the providers had a good practice of PCC.The Obstetric care providers’ practice scores range from 1 to 136 out of the possible maximum 136 points score. Despite their differences, all participants have practised at least a single component of the PCC practice question.

### Factors associated with the knowledge of obstetric care providers on preconception care

In this study, Midwives were two times more likely to have a good knowledge than nurses [AOR: 2.03, 95%CI: 1.09–3.77]. The likelihood of having good knowledge of PCC was three-fold higher among degree and above holders than those diploma holders [AOR: 3.11, 95%CI: 1.57–6.15]. Those OBCPs who read PCC guidelines or protocols from any source were two times more knowledgeable than those who never read PCC guidelines or protocols which are developed by other countries [AOR: 1.85, 95%CI: 1.09–3.12].OBCPs who were working in hospitals were two times more knowledgeable than those professionals who were working in health centres [AOR: 2.12, 95%CI: 1.1–3.8]. The other associated factor found in this study was, having training on HIV testing and management. Those OBCPs who had training on HIV testing and management were more than three-folds knowledgeable as those who did not have the training [AOR 3.51, 95%CI: 1.93–6.40]. OBCPs who work in a facility that had a library were two times more knowledgeable than those whose facilities had no library [AOR: 1.73, 95%CI: 1.04–2.85]. Those professionals who were working in facilities that had internet access were more than three folds knowledgeable as those whose facilities had no internet access [AOR: 3.45, 95%CI: 2.05–5.81] (Table 4).

**Table 4:** Binary and multivariate logistic regression analysis results of preconception care knowledge among obstetric care providers, West Shoa Zone, Oromia, Ethiopia, 2021.

Variables		OBCPs knowledge on PCC		COR(95%C.I)	AOR(95% C.I)	P-value
		Good	Poor			
Gender	Male Female	95(51.9%) 78(44.3%)	88(48.1%) 98(55.7%)	1.35(0.89–2.05) 1*	1.28(0.77–2.12) 1*	0.33
Profession	Doctor Nurse Midwife Health officer	23(67.6%) 31(35.2%) 95(56.5%) 24(34.8%)	11(32.4%) 57(64.8%) 73(43.5%) 45(65.2%)	3.84(1.6 5–8.91) 1* 2.39(1.40–4.07) 0.98(0.50–1.89)	1.30(0.44–3.82) 1* 2.03 (1.09–3.77) 0.4 7(0.21–1.05)	0.62 0.02** 0.06
Educational level	Degree and above Diploma	147(51.6%) 26(35.1%)	138(48.4%) 48(64.9%)	1.96(1.15–3.34) 1*	3.11(1.57–6.15) 1*	0.001**
Working institution	Hospital Health center	67(65.7%) 106(41.2%)	35(34.3%) 151(58.8%)	2.72(1.6 9–4.39) 1*	2.12(1.17–3.84) 1*	0.01**
Ever read PCC guidelines from any source	Yes No	120(57.4%) 53(35.3%)	89(42.6%) 97(64.7%)	2.46(1.60–3.80) 1*	1.85(1.09–3.12) 1*	0.02**
Training on HIV testing and management	Yes No	143(57%) 30(27.8%)	108(43%) 78(72.2%)	3.44(2.11–5.61) 1*	3.51 (1.93–6.40) 1*	0.00**
Training on providing alcohol or tobacco cessation service	Yes No	130(53.5%) 43(37.1%)	113(46.5%) 73(62.9%)	1.95(1.24–3.07) 1*	1.04(0.5–1.90) 1*	0.89
Presence of library in working health facility	Yes No	104(56.2%) 69(39.7%)	81(43.8%) 105(60.3%)	1.95(1.28–2.97) 1*	1.73(1.04–2.85) 1*	0.03**
Presence of internet access in working health facility	Yes No	115(59.6%) 58(34.9%)	78(40.4%) 108(65.1%)	2.74(1.78–4.21) 1*	3.45(2.05–5.81) 1*	0.00**
The perceived expectation on who should give PCC	All health care providers Selected health care provider	72(53.3%) 101(45.1%)	63(46.7%) 123(54.9%)	1.39(0.90–2.13) 1*	1.48(0.87–2.54) 1*	0.14
Opinion on which health facility should give PCC services	All health facility Selected health facility	61(58.1%) 112(44.1%)	44(41.9%) 142(55.9%)	1.75(1.11–2.78) 1*	1.35(0.76–2.41) 1*	0.29

*Reference,

** Significant at P-Value <0.05, COR- Crude Odds Ratio, AOR-Adjusted Odds Ratio, CI = Confidence Interval.

### Factors associated with obstetric care providers’ practice on preconception care

Participants’ knowledge of preconception care had no significant association with their level of practice. Obstetric care providers who were working in MCH [AOR: 3.90, 95%CI: 1.94–7.82] and Gynecologic OPD [AOR: 3.74, 95%CI: 1.69–8.24] had a more likelihood of PPC practice compared to OBCPs who was working in the labour& delivery ward. The likelihood of good practice of PCC was four-fold higher among obstetric care providers who did screening for RPL plan of clients than those who didn’t screen [AOR: 3.51, 95%CI: 1.76–6.98]. In addition, those professionals who ever read preconception guidelines and protocol from any source were two times more likely to have good practice of PPC than those who never read [AOR: 1.82, 95% CI: 1.03–3.23].

OBCPs who was working in a health facility that had internet access were three times more likely to have good practice than those OBCPs who were working in a health facility that did nothave an internet access [AOR:3.29, 95% CI: 1.82–5.95].OBCPs who possessed a perceived expectation that PCC should be given by all health care professionals had higher odds of PCC practice than those who perceived PCC should be given by selected professionals [AOR: 3.02, 95% CI:1.69–5.41)]. The odds of good PCC practice was two times higher among OBCPs who perceived PCC should be given in all health facility than those who perceived PCC should be given in selected health facility [AOR: 2.37,95% CI:1.29–4.33]. The likelihood of good practice of PCC was two times higher among obstetric care providers who had training on RPL plan screening and counselling than those who did nothave [AOR:1.77,95%CI: 1.01–3.10] (Table 5).

**Table 5:** Binary and multivariate logistic regression analysis results of preconception care practice among obstetric care providers, West Shoa Zone, Oromia, Ethiopia,2021.

Factors		OBCPs practice on PCC		COR (95.0%C.I)	AOR(95.0% C.I)	P-value
		Good	Poor			
Age	20–25	8(47.1%)	9(52.9%)	1*	1*	
	26–30	57(39.3%)	88(60.7%)	0.72(0.26–1.99)	1.20(0.36–3.94)	0.76
	31–35	32(33%)	65(67%)	0.55(0.19–1.57)	0.86(0.24–3.11)	0.82
	≥36	27(27%)	73(73%)	0.41(0.14–1.18)	0.76(0.20–2.95)	0.70
Working experience	>5 years	63(30.4%)	144(69.6%)	0.65(0.42–1.01)	0.56(0.29–1.07)	0.07
	≤5 years	61(40.1%)	91(59.9%)	1*	1*	
Working unit	Maternal child health care	55(50.9%)	53(49.1%)	5.23(2.89–9.47)	3.90(1.94–7.82)	0.00**
	Gynecologic OPD	28(41.8%)	39(58.2%)	3.62(1.85–7.05)	3.74(1.69–8.24)	0.001**
	Gynecologic ward	19(37.3%)	32(62.7%)	2.99(1.44–6.21)	2.23(0.96–5.18)	0.06
	Labor anddelivery ward	22(16.5%)	111(83.5%)	1*	1*	
Attitude	Favorable	93(36.5%)	162(63.5%)	1.35(0.82–2.21)	1.26(0.66–2.38)	0.47
	Unfavorable	31(29.8%)	73(70.2%)	1*	1*	
RPL plan screening	Screening	107(42.1%)	147(57.9%)	3.76(2.11–6.70)	3.51(1.76–6.98)	0.00**
	Not screening	17(16.2%)	88(83.8%)	1*	1*	
Ever read PCC guideline or protocol from any source	Yes	87(41.6%)	122(58.4%)	2.17(1.37–3.45)	1.82(1.03–3.23)	0.03**
	No	37(24.7%)	113(75.3%)	1*	1*	
Training on PCC consideration for clients with chronicdisease	Yes	88(40.4%)	130(59.6%)	1.97(1.24–3.14)	1.70(0.96–3.01)	0.06
	No	36(25.5%)	105(74.5%)	1*	1*	
Training on RPL plan screening and counseling	Yes	56(39.2%)	87(60.8%)	1.40(0.90–2.17)	1.77(1.01–3.10)	0.04**
	No	68(31.5%)	148(68.5%)	1*	1*	
Presence of internet access in working health facility	Yes	88(45.6%)	105(54.4%)	3.02(1.90–4.82)	3.29(1.82–5.95)	0.00**
	No	36(21.7%)	130(78.3%)	1*	1*	
Perceived expectation on who should give PCC	All health care professionals	67(49.6%)	68(50.4%)	2.88(1.83–4.53)	3.02(1.69–5.41)	0.00**
	The selected health care professional	57(25.4%)	167(74.6%)	1*	1*	
Opinion on which health facility should give PCC services	All health facility	55(52.4%)	50(47.6%)	2.94(1.8 3–4.73)	2.37(1.2 9–4.33)	0.005**
	Selected health facility	69(27.2%)	185(72.8%)	1*	1*	

*Reference,

** Significant at P-Value <0.05,COR- Crude Odds Ratio, AOR-Adjusted Odds Ratio, CI = Confidence Interval.

## Discussion

This study revealed that 48.2% [95%CI: 43.2–53.5] of OBCPs had a good knowledge of PCC. This finding is consistent with the studiesdone in Brazil (46.2%) [17], Nigeria 52.7% [18], Awi, Ethiopia 52% [12], and Wollo, Ethiopia(49.1%) [13]. However, it is higher than a study done in Hawassa(31%) [11]. This difference may be due to the concept of PCC was a new initiative at the time of the study, and the non-inclusion of PCC courses in pre-service training [19], and currently, the care was included in pre-service curricula of some programs. The present finding is lower than the studies done in TikureAnbessa Hospital Ethiopia 69.2% [15], Iran (88.3%) [20], Nepal (85.9%) [21], and South Africa (55%) [22]. This might be due to, unlike in Ethiopia PCC service existed and was implemented earlier, and also it was considered to be a part of integrated care and responsibility of providers so that this can increase the provider’s exposure to PCC in the case of Iran [23]. The possible reason for the study of Nepal and South Africa might be due to differences in sample size and sampling technique. The other possible explanation for the differences with the study of TikurAnbessa might be due to the study participants’ academic profile, being the largest teaching and referral hospital in Ethiopia and the current study was done in urban and more rural areas which intern affects the provider’s accessibility to information and updated knowledge.

This study revealed that 34.5% (95%CI: 29.5–39.6) of OBCPs had a good practice on PCC. This finding was lower than the studies done in Canada (78.4%) [24] and Netherlands 82% [25]. This difference may be due to PCC service being a new initiative that is yet to be introduced in the health care system of Ethiopia and in those countries dedicated PCC service unit was established for the provision of PCC [10]. The other reason might be, unlike those countries, Ethiopia did notyet develop PCC practice guidelines rather preconception guidance was usually contained within maternity guidelines [19]. However, the current finding was higher than the study conducted at TikurAnbessa hospital where is 19.2% of residents had a good level of preconception care practice [15]. This difference may be due to the samplesize difference.

In this study, participant’s profession was associated with obstetric care providers’ knowledge about PCC. Midwives were two times more likely to have agood knowledge of PCCcompared to nurses. This finding is in line with the study conducted in Wollo, Ethiopia [13]. The knowledge gap might be due to, unlike nurses, midwives might have chances to be exposed to preconception care during their pre-service training [26] and their working departments being linked to the components of PCC.

The likelihood of having good knowledge of PCC was three-fold higher among degree andabove holders compared to diploma holders. This finding was supported by a study conducted in Nepal [21]. This might be due to the educational curriculum differences.

Those OBCPs who read PCC guidelines or protocol from any source were two times more knowledgeable than those who never read PCC guidelines or protocol. This finding was supported by the studies done in Wollo, Ethiopia [13] and Hawassa, Ethiopia [11]. This finding is considerable in that getting access to different guidelines and procedural documents that guide PCC will enable providers to be informed and to know the components of PCC services.

OBCPs who were working in the hospitals were two times more knowledgeable than those professionals who were working in the health centre. This finding is also in line with a study done in Hawassa [11]. This might be due to the presence of different higher specialized care; they had a chance of exposure to different clinical cases than health centres.

The other factor found in this study is having training on HIV testing and management. Those OBCPs who had training on HIV testing and management were more than three-folds knowledgeable than those who did nothave the training. The finding was in line with the studies conducted in Nepal [21] and Awi, Ethiopia [12]. This is literal in that when professionals’ get in-service training they will get updated knowledge and the HIV training manuals included preconception care components [27].

OBCPs who work in an institution that had library and internet access were two times and more than three-fold knowledgeable respectively. This finding was supported by a study done in Wollo, Ethiopia [13]. This is literal in that, HCPs can access those guidelines, different medical-related books, and manuals for reading, gathering, and updating information therefore this can intern will increase their knowledge [28,29].

The likelihood of good practice of PCC was fourfold higher among obstetric care providers who did screening for RPL plan of clients than those who did notscreen; this finding was in line with a study done in Hawassa, Ethiopia [14]. This is possible in that requesting women’s RPL plan could serve as an opening wedge to initiate several evidence-based pre-pregnancy care interventions [30].

Those professionals who ever read preconception guidelines and protocol from any source were two times more likely to have agood practice than those who never read. This finding is also supported by studies done in the Netherlands and Australia [31–33]. This is possible in that knowing guidelines about PCC will serve as guidance and motivate to give effective PCC services.

OBCPs that possessed a perceived expectation that PCC should be given by all health care professionals had odds of good PCC practice by three-fold than those who perceived PCC should be given by selected professionals. This finding was in line with a study done in Hawassa [14]. This is possible in that having this opinion makes the professional himself take responsibility to give the care.

### Strength and limitations of the study

This study considers all health care providers responsible for the provision of preconception care. However, it has some limitations: Firstly, this study was not supported with observational studies for the assessment of preconception care practice. Hence, we cannot validate their actual performance. Secondly, there might be the possibilities of recall and social desirability bias.

## Conclusions

This study found that more than half of obstetric care providers’ had poor knowledge and practice of preconception care. Respondents’ profession, educational level, type of working institution, ever read PCC guidelines from any source, having training on HIV testing and management and institutions having library andinternet access were associated with the providers’ knowledge. On the other hand, participants working unit, training on RPL plan screening andcounselling, RPL plan screening, ever read PCC guidelines or protocol from any source, presence of internet access in working health facilities, perceived expectations on who should give PCC, and opinion on which health facility should give PCC services were associated to the providers’ good practice. Provision of training, carrier development, and installation of internet and library services in working facilities is recommended.

## Supporting information

S1 Data set (SAV)

S1 Questionnaire (PDF)

## Abbreviations

APO: Adverse Pregnancy Outcomes
PCC: Preconception Care
OBCPs: Obstetric Care Providers
HCP: Health Care Provider
WHO: World Health Organization
