# Supplementary material for: Knowledge, attitude, and practice of preconception care and associated factors among obstetric care providers working in public health facilities of West Shoa Zone, Ethiopia: A cross-sectional study
Source: PLoS One. 2022 Aug 1;17(8):e0272316. doi: 10.1371/journal.pone.0272316 (PMC9342760; doi:10.1371/journal.pone.0272316)
Supplement: S1 Questionnaire — (PDF) [file pone.0272316.s002.pdf]

Questionnaires

Instruction: Please read the following questions listed and tick in the box of the best option you choose and write your answer on the space provided to some of the questions.

PART ONE: Socio-demographic, professional, and work-related questions

1.1. Gender 1.□ Male 2.□ Female

1.2. Your age in years _______ year

1.3. Marital status

1.□ Single 2. □ Married 3. □ Divorced 4. □ Widowed 5. □ Living together

1.4.Religion 1.□ orthodox 2. □ Protestant 3.□ Muslim 4 □ Catholic 5.□ other_____

1.5. Profession

1. □ Medical Doctor 3.□Midwife

2. □ Nurse 4.□ Public Health officer 5.□ Other_________________

1.6.Year of experience/ ___________ year

1.7. Maximum educational level attended with your health profession

1. □ Diploma 3. □ general practitioner/medical doctor

2. □ Bachelor of Science (BSc) 4.□ Specialty 5.□ PhD

1.8. In which department you are currently working? ______________________

1.9. Monthly salary paid in **Ethiopian birr ________** Birr/month

1.10. How many patients do you manage per day on average? ______ patients/day

PART TWO: Preconception Care (PCC) Knowledge related questions.

Instruction: Please read the following questions listed and tick in the box of the best option you choose.

	Items	Yes	No	Don't know
2.1	The eligible clients for preconception care (PCC) include all adolescents and reproductive-aged individuals	1□	2 □	3□
2.2	To be effective PCC should start four weeks before conception	1□	2 □	3□
2.3	Periodontal disease is a risk factor for adverse pregnancy outcomes (APO)	1□	2 □	3□
2.4	Women with BM I ≤ 18.4 planning pregnancy are at risk of developing APO	1□	2 □	3□
2.5	All women of reproductive age should take 0.4 mg (400 mcg) of folic acid daily.	1□	2 □	3□
2.6	If yes for Q 2.5, when should the women need to start taking folic acid?	1.3months prior to pregnancy<br>2. after confirmed pregnancy		
2.7	The recommended routine pre-conceptual laboratory tests include, Hct, HIV,HBV, and RPR or VDRL tests	1□	2 □	3□
2.8	Preconception genetic counseling and screening include recommending carrier screening tests for the client with sickle cell hemoglobinopathies	1□	2 □	3□
2.9	Isotretinions, Valproic acid, and Warfarin are medications poses teratogenic effects requiring preconception modification	1□	2 □	3□
2.10	Early identification and treatment of diseases like depression, seizure disorder, and phenylketonuria during the preconception period reduce the occurrence of APO	1□	2 □	3□
2.11	The recommended test that guarantees good preconceptional blood sugar control for a woman with pre-gestational diabetes is random blood sugar (RBS) test	1□	2 □	3□

2.12	Recommending regular exercise is an important PCC counseling point. Thus, women planning pregnancy should aim 30 minutes of moderate exercise 5 days a week.	**1□**	**2 □**	3□
2.13.	Women planning pregnancy should be advised to delay pregnancy until reducing drug, alcohol and tobacco use	1□	2 □	3□
2.14.	A clinician attending clients with previous caesarian section (C/S) should advise the client to delay the next pregnancy for at least 18 months before the next conception	1□	2 □	3□
2.15.	Infertility screening and management is not the concern of PCC	1□	2 □	3□

PART THREE: Preconception Care (PCC) Practice related questions

Instruction: Please read the following questions listed and tick in the box of the best option you choose and write your answer on the space provided to some of the questions

1. Do you ask for the reproductive life plan (RPL) of clients attending to your day to day practice? 1. □ Never 2. □ Rarely 3.□ Sometimes 4.□ Often 5. □ always

2.If you at least rarely ask for Reproductive life plan (RPL), whom do you asking? (Select all that can apply and add if you have more)

1. □ Adult males (≥ 18 years)
3. □ Adult females (18 – 49 years)
2. □ Adolescents and Teens (12-18 Years)
4.□ Indicate if other ___________________

Do you in your practice, for a client, give counseling about issues listed from Question 301-313 in the past 3 months of period?

	About…	**Never**	**Rarely**	**Sometimes**	**Often**	**Always**
301.	Family planning methods	1 □	2 □	3 □	4 □	5 □
302.	Pregnancy spacing	1 □	2 □	3 □	4 □	5 □
303.	Physical exercise	1 □	2 □	3 □	4 □	5 □
304.	Body weight	1 □	2 □	3 □	4 □	5 □
305.	Nutrition	1 □	2 □	3 □	4 □	5 □

306.	Alcohol tobacco, and psychoactive substance use	1 □	2 □	3 □	4 □	5 □
307.	Multivitamin containing Folic acid	1 □	2 □	3 □	4 □	5 □

308.	Importance of maintaining good control of any preexisting medical conditions before conception	1 □	2 □	3 □	4 □	5 □
309.	Importance of screening for STIs/HIV	1 □	2 □	3 □	4 □	5 □
310.	Dangers of prescribed and non-prescribed medication use	1 □	2 □	3 □	4 □	5 □
311.	Environmental hazard & toxins	1 □	2 □	3 □	4 □	5 □
312.	Preventive vaccines	1 □	2 □	3 □	4 □	5 □
313.	The importance of inviting partner for preconception counseling, risk screening, and management	1 □	2 □	3 □	4 □	5 □

Ple**ase read the following questions listed and tick in the box of the best option you choose. Did you in your practice, for a client, conduct the following preconception health assessment in the past 3 months (Question 314-325) to find preconception health risk factors?**

		**Never**	**Rarely**	**Sometime**	**Often**	**Always**
314.	Past Obstetric & Gynecologic history	1 □	2 □	3 □	4 □	5 □
315.	Past medical and surgical history	1 □	2 □	3 □	4 □	5 □
316.	Genetic history or family pedigree	1 □	2 □	3 □	4 □	5 □
317.	History of dental care/checkup	1 □	2 □	3 □	4 □	5 □
318.	Social history particularly lifestyle behaviors	1 □	2 □	3 □	4 □	5 □
319.	Exposure to environmental toxins and contaminants	1 □	2 □	3 □	4 □	5 □

320.	Pharmacologic history	1 □	2 □	3 □	4 □	5 □
321.	Nutritional assessment particularly BMI	1 □	2 □	3 □	4 □	5 □
322.	Psycho-social assessment	1 □	2 □	3 □	4 □	5 □
323.	Physical examination	1 □	2 □	3 □	4 □	5 □
324.	Employment history	1 □	2 □	3 □	4 □	5 □
325	Vaccination status	1 □	2 □	3 □	4 □	5 □

Please read the following questions listed and tick in the box of the best option you choose. Do you in your practice, for a client planning pregnancy, carryout the following intervention either yourself or by referring or transferring the client to other department/health facility where the client/s get services indicated in the table below?

		**Never**	**Rarely**	**Sometimes**	**Often**	**Always**
326.	Folic acid supplementation/prescription	1 □	2 □	3 □	4 □	5 □
327.	Substance use cessation. Eg. alcohol, cigarette, or other drugs	1 □	2 □	3 □	4 □	5 □
328.	Select safe medication or substitute the existing with a safe one	1 □	2 □	3 □	4 □	5 □
329.	Ordering/checking routine preconception lab investigations	1 □	2 □	3 □	4 □	5 □
330.	Diagnosing & managing acute or chronic preconception risk Conditions	1 □	2 □	3 □	4 □	5 □
331.	Controlling existing pre-gestational chronic diseases	1 □	2 □	3 □	4 □	5 □
332.	Vaccination of client as per the national protocol	1 □	2 □	3 □	4 □	5 □

333.	Pregnancy confirmation	1 □	2 □	3 □	4 □	5 □
334.	Linking client to other relevant department or organization	1 □	2 □	3 □	4 □	5 □
335.	Provider initiated HIV testing and counseling (PIHTC)	1 □	2 □	3 □	4 □	5 □

PART FOUR: HP's levels of Agreement/Disagreement on selected PCC issues to assess Attitude towards Preconception. **Instruction**: Please read each questions listed from Qn # 401- 409) and respond to each questions by mentioning your level of agreement or disagreement by ticking the box of the options indicated as 1 =>*StronglyDisagree*, 2=>*Disagree*, 3=>*Neutral or Undecided*, 4 =>*Agree*, and 5=>*Strongly disagree*

		**Strongly Disagree**	**Disagree**	**Undecided**	**Agree**	**Strongly Agree**
401.	Omission of preconception care leads to an irreversible damage to the fetus	1 □	2 □	3 □	4 □	5 □
**402.**	PCC provides a greatest opportunity to optimize couples health particularly women's health before conception	1 □	2 □	3 □	4 □	5 □
403.	Providing PCC service to developing countries like Ethiopia is a luxury service	1 □	2 □	3 □	4 □	5 □
404.	In developing country like Ethiopia, the focus of PCC should not be directed to healthy people but for people with infectious disease like HIV and HBV	1 □	2 □	3 □	4 □	5 □
**405.**	Providing PCC is not within the scope of my professional responsibility and accountability.	1 □	2 □	3 □	4 □	5 □
406.	Due to the presence of other competing demands, providing PCC is not the priority intervention I should provide.	1 □	2 □	3 □	4 □	5 □
407.	Preconception care should be given for all healthy and sick individuals including those presented with critical and emergency condition.	1 □	2 □	3 □	4 □	5 □
408.	All healthcare providers (professionals) can easily integrate the elements of PCC in their daily practice to all eligible	1 □	2 □	3 □	4 □	5 □

	individuals whom they are caring					
409.	Pre conception health is part of the reproductive and human right issue to which the health professional is responsible either for omission or commission of PCC	1 □	2 □	3 □	4 □	5 □

PART FIVE: Additional factors associated with Preconception practice

Please read the following questions listed and tick in the box of the best option you choose.

Have you taken training on or thought about topics listed from (Select all that can apply)Qn # 501 - 508?

		Yes during my stay at University College (Pre-service training)	Yes as an in-service training	Never ever get the training	Don't remember
501.	Reproductive life plan screening & brief counseling	1 □	2 □	3 □	4 □
502.	the importance of increasing public awareness Preconception health &PCC	1 □	2 □	3 □	4 □
503.	how to conduct preconception risk assessment	1 □	2 □	3 □	4 □
504.	how to provide preconception educational & counseling	1 □	2 □	3 □	4 □
505.	how to manage identified preconception risk factors	1 □	2 □	3 □	4 □
506.	About HIV/AIDS Testing and management (E.g. PMTCT, PIHCT,VCT, or ART)	1 □	2 □	3 □	4 □
507.	PCC considerations for clients with other chronic diseases	1 □	2 □	3 □	4 □
508.	how to provide alcohol or tobacco cessation service	1 □	2 □	3 □	4 □

PART SIX: Regarding actual & potential access to resources. Please read the following questions listed and tick in the box of the best option you choose.

		Yes	No	
601.	Do you have access to an internet	□1	□2	If "No" go to Qn#603
602.	From where do you get internet access? **(Select all that can apply)** □ 1. office or library □ 2 From an internet cafe □ 3 From hotel WIFI service □ 4 mobile phone			
603.	Do you use your Smartphone(SP) to share e-resources from others	□1	□2	□ 3 I Don't have SP
604.	Does your institution have a library?	□1	□2	
605.	Does your institution have a policy and procedural document guiding PCC?	□1	□2	□ 3 I Don't know
506.	Did you, so far, get or see any PCC gridline or protocol from any source?	□1	□2	
607.	Have you ever seen national PCC guidelines or protocols prepared by FMOH (Federal ministry of health)?	□1	□2	□ 3 I Don't know
608.	Have you seen any HP practicing PCC in your facility?	□1	□2	
609.	Are you willing to incorporate elements of PCC in your daily practice?	□1	□2	□ 3 Undecided
610.	Do you want training on PCC?	□1	□2	

611. Whom do you recommend to provide preconception care (Select all that can apply & add if more)

1. □ All Specialist Doctors
2.□ All General Practitioners
3. □ All Nurses
4. □ All midwives
5. □ Health 0fficers
6.□ Health Extension workers

7.Others.__________________

612. In which facility should preconception care service be given (select all that can apply and add if you suggest other)

1. □ Health center
2. □ Hospital
3. □ Other. ______________________________

613. Have you received client request for preconception care counseling and consultation in the past three months?

614. If you provide PCC in the past three months, for how many times you provide PCC? ____ times in the past three months (please write it in number)

Thank you for participating in this study!!!

<table>
<tr><th colspan="3">General Information (To be filled by Research Assistant and Supervisors)</th></tr>
<tr><td>Health Facility Name</td><td></td><td>Are all pages checked for availability and completeness?</td></tr>
<tr><td>Date the questionnaire was completed</td><td>______/________/ 2021</td><td rowspan="3">Remark by Data collector</td></tr>
<tr><td>Data collector name
Signature</td><td>________________________
___________</td></tr>
<tr><td>HP’s Department</td><td>______________________
_____</td></tr>
<tr><td>Codes/PHI – UCSC(Eg. 00/00/ - 000)</td><td>_________/__________/ -
_____________</td><td rowspan="3">Remark by Supervisor</td></tr>
<tr><td>Name of the Supervisor
Signature</td><td>________________________
___________</td></tr>
<tr><td>Date checked by supervisor</td><td>_____/_______/ 2021</td></tr>
</table>
