# Supplementary material for: Knowledge, attitude, and practice of preconception care and associated factors among obstetric care providers working in public health facilities of West Shoa Zone, Ethiopia: A cross-sectional study
Source: PLoS One. 2022 Aug 1;17(8):e0272316. doi: 10.1371/journal.pone.0272316 (PMC9342760; doi:10.1371/journal.pone.0272316)
Supplement: Attachment — Submitted filename: PONE-D-22-10177_SG.docx [file pone.0272316.s003.docx]

Reviewer’s Comment

**Title**:

Knowledge and practice of preconception care and associated factors among obstetric

care providers working in public health facilities of West Shoa Zone, Ethiopia: A cross-sectional study

General Comment:

In general, it is a well design study, conducted in a way scientifically and technically sound methodology. Appropriate data analysis method was employed, and figures are consistent through the document. Nevertheless, this manuscript needs editorial revision and minor changes to the abstract and discussion to make it clear and more attractive for the reader.

Detail line by line comments are provided below.

Title:

L112-118: the Data collection tools and procedures indicate that the study tool has five sections(demographic information, knowledge, attitude and practice, and associated factors of preconception care questions). The proceeding sentence also shows that you have assess attitude as well. Therefore, attitude should be included in the title, keywords, and result sections.

Since the data collection tool(questionnaire) is not provided, I couldn’t verify the actual questions that address the attitude section. Please, provide the study materials with your revised manuscript.

**Abstract**

L27-33: Please paraphrase the result section of the abstract.

- L27-30: has too many conjunctions “and/&” and it is unclear.
- Select the most relevant findings to be included in the abstract.

L32: "doing screening for reproductive life plan"

- This is a very specific task. I suggest using professional category or the department instead of pointing out a very specific task. Also, it would be much better if the result can be presented in carrier-wise like diploma, degree, midwives, physicians, and other gyn & obs care providers in the selected health facilities.

Keywords:

Add attitude if providers’ attitude was assessed, otherwise the attitude assessment questionnaire should be removed from the tool(See comment L112-118).

Methods

L98: “ *the first two specific objectives”* - Please mention each objective here.

L106-107: Please, paraphrase as *“ The sample was allocated to each stratum proportionally based on the number of health care providers working at the selected hospitals and health centers”.*

L114 - Indicates that knowledge, attitude, and practice preconception care of participants were assessed. See my comments above.

L122 -124: Data quality assurance is beyond a questionnaire that includes data collection, data processing and data management. So, please paraphrase as

*"To assure quality of the study, data collectors and supervisors were given two days training about the study materials and data collection procedures. The study tool was pre-tested among ...... "*

L127: “… *Cronbach's α tests which were 0.802 & 0.97 for knowledge and practice, respectively.”*

L172 - “1 -to 12” correct as 1 to 12

L174 -	“ 7-to 136” correct as 1 to 136

L175-176: I don't think this sentence is important. The above sentence showed that how many of them perform good practice based on your classification of good and poor.

Otherwise, you can explain as "only three participants were found to perform all the expected good practice on preconception care.... It is better focus on the meaning than the numbers.

L187: I think the health centers are also public. This can mislead to a comparison of public and non-public sectors.

Discussion and conclusion

- Please include strengths and limitations of this study.

Strength and limitation of the study should be included.
